# In Defence of Dr. S. C. Barnum

**Published:** 1884-01

**Authors:** G. A. Mills


ARTICLE III.

### IN DEFENCE OF DR. S. C. BARNUM.

In June last I met Dr. S. C. Barnum of Rubber Dam fame, in the S. S. White up-town house, New York. I was attracted particularly to him by his decidedly changed physical condition. I found him a picture of a distressed victim of nervous exhaustion; he told me he had not been able to operate at the chair for some months, but was doing something in artificial work. After a little general conversation, he said to me, " Dr. Mills, were you not present at the meeting of the Old New York Society held in Cooper Union, the evening my uncle, Dr. Clewes, introduced the use of the Rubber Dam and presented it to the dental profession as coming from me? This was, I think, in 1866." I replied I was, and that in 1874 I read a paper before the American Convention at Saratoga; Subject, " What next ?" "In this I gave the history of the introduction of your Dam, and the manner in which it was received, &c." I said, " and after the enthusiasm had subsided, Dr. George E. Hawes arose in his peculiar style, and quaintly asked, "What next." This article is in print in one of our Dental Journals. "Well," Dr. Barnum says, "Dr. Mills, I am exceedingly troubled that an effort is being zealously used to take all the *credit* from me and to place me in a very unenviable position before the whole profession, branding me as dishonest, for as you know, I have been the recipient of several tangible and valuable testimonials." I said to him, "you need have no fear, the profession will stand by you. No man can afford to promulgate such a claim of priority, for it comes too late."

He says, "Do you know that Dr. Frank Abbott, President of the New York Dental College, is teaching the students that Dr. Laroche, Sr., is the *originator* of this Dam? Dr. Laroche is the Vice President of the College." I replied to him, "You be patient, and if these men have the audacity to make this claim, you will find your friends will come to your rescue. As I live, you can count on me, no matter *what* can be brought as proof on their part. That they should allow *all* these years to pass, and be *all* the time amongst us knowing *all* that has been done, and that you were being made the receiver of so many favors and not a lisp made to disapprove your claim, will strike every high and fair-minded member of our profession as a great injustice, and they will say with one acclaim, 'Too late, Too late.' I told Dr. Barnum it would produce such a tidal wave of disapproval that no man or men could afford to face. I left him with this prediction, that they would never *dare* to uncover such claim, for I did not take either of these men to be foolish or crazy." My prophecy has not proved true.

At the meeting of the First District Dental Society, held November 6th, 1883, the subject of loose teeth historically considered, was down on the bulletin as the subject for the evening, but to the surprise of all, Dr. Laroche, Sr., presented a paper, claiming to be the originator of the Dam, and read affidavits as proofs. His date is fixed in 1857, some ten years prior to Dr. Barnum.

I will not attempt to describe the very apparent impression that was produced on those present. All kinds of imprecations were distinctly heard murmuring from all parts of the room. Dr. Abbott made an attempt to defend Dr. Laroche's claim by saying that at the time Dr. Barnum's claim was made known he did not think much about it, and as it was not very enthusiastically received, he let it pass, &c. To say I have a feeling of astonishment that I find no language to express, will be only saying what will be echoed from every nook of our profession where this great and blessed auxiliary has been demonstrated. I do not propose to discuss this matter, but leave it in the minds of our whole profession, "Who will deal justice to whom justice belongs," "Honor to whom honor is due," with a suggestion which presents itself forcibly to my mind. 'What will we do about it?" As we here all know Dr. S. C. Barnum to be a man of modest pretensions and an upright member of our profession who has gone in and out before us all these years, quietly and unobtrusively, and we can but feel that to have his claim called in question at this late date, and considering all the publicity given it, and also considering his quiet and polite demeanor, his failing health, it is only kind and just to give him our sincere and hearty co-operation in maintaining more tenaciously the need of praise already accorded to him. I feel that to italicise these expressions is not enough; but it can be done in a more tangible and practical manner. Let us one and all enclose to him a dollar Postal Note, with our own words of encouragement and praise. By this we will be putting flowers on his home mantlepiece that shall shed a grateful fragrance, helping him to make his last days his best. Dr. Barnum is not over-supplied with this world's goods. His opponents have no need.

G. A. MILLS

DR. S. C. BARNUM "Address"
104 WEST 45TH STREET,
New York City, New York.

